# The Impact of a Multi-Level Multi-Component Childhood Obesity Prevention Intervention on Healthy Food Availability, Sales, and Purchasing in a Low-Income Urban Area

**DOI:** 10.3390/ijerph14111371

**Published:** 2017-11-10

**Authors:** Joel Gittelsohn, Angela C. Trude, Lisa Poirier, Alexandra Ross, Cara Ruggiero, Teresa Schwendler, Elizabeth Anderson Steeves

**Affiliations:** 1 Global Obesity Prevention Center, Department of International Health, Johns Hopkins Bloomberg School of Public Health, Johns Hopkins University, 615 N. Wolfe Street, Baltimore, MD 21205, USA; atrude1@jhu.edu (A.C.T.); lpoirie4@jhmi.edu (L.P.); aross37@jhu.edu (A.R.); teresarose199@gmail.com (T.S.); 2 Center for Human Nutrition, Department of International Health, Johns Hopkins Bloomberg School of Public Health, Johns Hopkins University, 615 N. Wolfe Street, Baltimore, MD 21205, USA; 3 Center for Childhood Obesity Research, Department of Nutritional Sciences, Pennsylvania State University, University Park, PA 16802, USA; cfr8@psu.edu; 4 Department of Nutrition, University of Tennessee, 1215 W. Cumberland Ave., Knoxville, TN 37996, USA; eander24@utk.edu

**Keywords:** food availability, food purchasing, African American, food environment, childhood obesity, urban

## Abstract

The multifactorial causes of obesity require multilevel and multicomponent solutions, but such combined strategies have not been tested to improve the community food environment. We evaluated the impact of a multilevel (operating at different levels of the food environment) multicomponent (interventions occurring at the same level) community intervention. The B’more Healthy Communities for Kids (BHCK) intervention worked at the wholesaler (*n* = 3), corner store (*n* = 50), carryout (*n* = 30), recreation center (*n* = 28), household (*n* = 365) levels to improve availability, purchasing, and consumption of healthier foods and beverages (low-sugar, low-fat) in low-income food desert predominantly African American zones in the city of Baltimore (MD, USA), ultimately intending to lead to decreased weight gain in children (not reported in this manuscript). For this paper, we focus on more proximal impacts on the food environment, and measure change in stocking, sales and purchase of promoted foods at the different levels of the food system in 14 intervention neighborhoods, as compared to 14 comparison neighborhoods. Sales of promoted products increased in wholesalers. Stocking of these products improved in corner stores, but not in carryouts, and we did not find any change in total sales. Children more exposed to the intervention increased their frequency of purchase of promoted products, although improvement was not seen for adult caregivers. A multilevel food environment intervention in a low-income urban setting improved aspects of the food system, leading to increased healthy food purchasing behavior in children.

## 1. Introduction

Obesity and other diet-related chronic diseases have emerged as the greatest contributors to morbidity and premature mortality across the globe [1,2]. However, no single solution to this problem exists. The causes of obesity are multifactorial and require multiple coordinated actions to address this important public health problem [3], yet, most interventions and policies tested to date have focused on single solutions. 

In the past decade, there has been growing interest in multilevel and multicomponent interventions [4,5]. Some of these early trials have shown positive impact in reducing obesity prevalence [6,7,8]. To date, none of these previous multilevel obesity interventions have sought to modify levels of the community food environment simultaneously, and in a coordinated fashion. The community food environment is composed of many different food sources [9], which vary by number, type, location, and accessibility [10,11].

From 2014 to 2016, we implemented a multilevel intervention to improve the community food environment in low-income underserved areas of the city of Baltimore, called B’more Healthy Communities for Kids (BHCK) [12]. This group-randomized controlled trial sought to test multiple hypotheses, including the potential for impacting: (i) the food distributor (wholesaler) to improve stocking of healthier foods available to food retailers; (ii) the food retailer (corner stores and carryouts) to improve community food access and availability; and (iii) the consumer (children and their families) to improve healthy food purchasing. In this paper, we will test the hypothesis that BHCK intervention successfully changed the community food environment, resulting in improved purchasing of healthy food choices by low-income predominantly African American children and their adult caregivers. This paper has the following specific aims:

(1) To evaluate the impact of the BHCK multilevel intervention on sales of promoted foods at the wholesaler level.
(2) To evaluate the impact of BHCK on stocking and sales of promoted foods in corner stores and carryouts.
(3) To assess the impact of BHCK on adult caregiver and child healthy food purchasing.

## 2. Materials and Methods

### 2.1. Setting and Study Design

**Figure 1:**
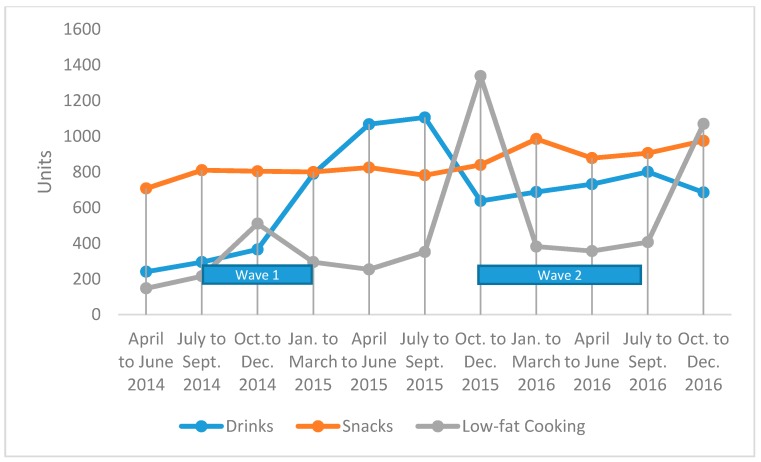
Sales of BHCK promoted drinks, promoted snacks, and promoted low-fat cooking ingredients during wave 1 and wave 2 of the BHCK wholesaler intervention.

B’more Healthy Communities for Kids (BHCK) was a multilevel, multicomponent randomized controlled trial that aimed to increase the demand for and access to healthy and affordable foods by way of multiple, coinciding interventions carried out at the individual, youth leader, corner store and carry-out, wholesale, and policy levels. The BHCK trial was informed by the social cognitive theory and the social ecological model. A detailed description of the BHCK intervention is provided elsewhere [12]. The intervention was implemented in two waves (Figure 1). Each wave of the BHCK intervention was implemented in seven low-income food desert zones (with seven comparison zones), where the nucleus of each zone was a community recreation center. Zone eligibility criteria for the trial were: (1) a predominantly African American (>50%) population; (2) minimum of five small food sources (<3 aisles, no seating) nearby; (3) having a recreation center; and (4) fitting the definition of a Baltimore food desert (median household income at or below 185% of the Federal Poverty level, >30% of households lacking access to a vehicle, and 1/4 mile from a supermarket [13]. 

Wave 1 occurred between July 2014–February 2015; and wave 2: November 2015–August 2016. Each wave of the BHCK trial was implemented in three phases, including: healthy drinks, healthy snacks, and healthy cooking methods, while the second wave had an additional reinforcement phase (Table 1). 

At the wholesaler level, managers were asked to stock healthier products, such as whole grain products, fruits and vegetables, and low-fat, low-sugar snacks and beverages (BHCK promoted items) [14]. Promoted foods were selected on the basis of formative research in low income areas of Baltimore city with adolescents, parents, small food source owners and local wholesalers [15]. Most promoted foods and beverages selected were available year-round. In corner stores and carryouts, owners were incentivized to stock and/or prepare foods using BHCK promoted items [14,16]. BHCK products were promoted in the stores through posters, shelf labels and signage. In addition, customers were exposed to these new products during educational interactive sessions conducted by BHCK interventionists in the stores. Customers tried food samples, received educational handouts and giveaways, and information detailing the health benefits of each food or beverage item. Concurrently on social media, educational materials and recipes were reinforced through posts on Facebook, Instagram, and Twitter and through text messages sent to adult caregiver participants [17]. In recreation centers, youth leaders (Baltimore college and high school students) taught lessons to children (10–14 years old) on nutrition topics and led cooking classes, such as making quesadillas with whole wheat tortillas and low fat cheese [18,19]. Each component reinforced and increased the exposure and dose of the intervention received by community members in the intervention zones. 

### 2.2. Measurements

#### 2.2.1. Wholesaler

Product movement and sales data of a subset of 19 BHCK promoted food items were collected from the one participating wholesaler. The data set included the quarterly movement and sales (from April 2014 to December 2016) of 49 wholesaler Universal Product Codes (UPCs) which reflected different variations (flavors, sizes, packaging) of the selected promoted foods. The 19 promoted foods selected were chosen by the research team to capture items which were promoted most heavily in the different phases of the corner store and carryout interventions. 

#### 2.2.2. Corner Stores & Carryouts Stocking and Sales

A Healthy Food Availability Index (HFAI) was created to assess changes in stocking and availability of different promoted foods in corner stores (*n* = 55) and carryouts (*n* = 30). During the two intervention waves, we completed monthly environmental assessment forms which documented the different food stocked and sold at each corner store via direct observation. Our environmental assessment form was adapted from the Nutrition Environment Measures Survey in Stores (NEMS-S) [20,21]. 

For the corner store HFAI, one point was awarded for the presence of each promoted food such as whole wheat bread or reduced fat cheese with a greater score indicating higher availability of healthy foods. Another scale was created for the number of fruit and vegetable varieties available at a store. If a store stocked between one and three varieties of fruit, they received 1 point. Stocking 4–6 varieties earned 2 points, 7–10 varieties earned 3 points, 11–25 varieties earned 4 points and finally, if a store had over 25 varieties of fruit, it earned 5 points. A similar scale was used to award points for vegetable varieties. The fruit and vegetable scale was then added to the rest of the points earned to create an overall HFAI score.

Subscores were created to match the themes of each intervention phase including healthy beverages, healthy snacks, and cooking. The combined subscores represent the total availability of the promoted foods from each intervention phase.

The carryout HFAI was developed similarly to the corner store HFAI. We conducted monthly carryout environmental assessments throughout the intervention period (wave 1: six intervention and eight comparison carryouts; wave 2: 10 intervention and six comparison carryouts). Each promoted food item or option, such as low fat choices for deli meat or grilled options for fresh proteins, present in the carryout or on the menu received one point. 

Sales and pricing data from each corner store was collected using the store impact questionnaire (SIQ), which was performed at baseline and post-intervention. The price of each item was collected through observation or interview with the owner/manager when not posted, and reported seven-day sales recall of each product was collected via interview with the owner/manager using a previously tested approach [22]. Small store owners in Baltimore do not keep electronic or written records of sales, making recall the only viable approach. The average of total sales was calculated, along with sub-totals for the themes of each intervention phase. 

#### 2.2.3. Child-Caregiver Dyads

A sample of adult caregiver and child dyads was recruited at each recreation center and nearby corner stores in a 1.5-mile zone buffer. Household eligibility criteria included: (1) being ≥18 years and a caregiver of at least one child in the age range of 10–14 years; (2) having lived in the same location for at least one month; and (3) does not anticipate moving in the next 2 years [12]. 

Baseline data were collected from June 2013 to June 2014 (wave 1) in a total of 299 caregiver-child dyads, and from July to November 2015 (wave 2) in 235 caregiver-child dyads [12]. Post-evaluation was conducted from April 2015 to November 2015 (wave 1) and from August 2016 to January 2017 (wave 2). A total of 385 children and 387 caregivers completed all baseline, follow-up and exposure assessments and were eligible for this analysis. Ninety percent of the sample self-identified as African American (Table 2). 

Healthy Food Purchasing Scores were developed from the Adult Impact Questionnaire (AIQ) [23], and from the Child Impact Questionnaire (CIQ) conducted with children [24] at baseline and follow-up. For the household food purchasing behavior, we asked the primary caregiver to report the number of times they purchased or got food from different food sources in the previous 30 days from the interview date (e.g., “How many times did you get these foods?”). A list of 31 BHCK promoted healthier food and beverage items was provided: 1% or skim milk, yogurt, diet soda or diet energy drinks, water, 100% fruit juice, sugar free drinks, unsweetened tea, fresh fruits such as apples, oranges, bananas, frozen fruit, fresh and frozen vegetables, canned tuna in water, dried beans, low sugar, high fiber cereals, 100% whole wheat bread, plain hot cereal, pretzels, baked chips, reduced-fat chips, dried fruit, nuts or seeds, reduced fat butter or margarine, cooking spray, lite mayonnaise. For each item the caregiver reported purchasing, we assigned a score of 1, and a 0 otherwise. Score ranged from 4 to 30 points, mean 19.1, SD: 4.8, Cronbach’s alpha: 0.74. 

For the child food purchasing behavior, we asked respondents to report the number of times they purchased or got food from different food sources in the previous seven days from the interview date (e.g., “How many times did you get these foods?”). A list of 34 BHCK promoted healthier food and beverage was provided: 1% or skim milk, diet soda, water, 100% fruit juice, sugar free drinks, fruit flavored water, unsweetened tea, fresh fruits such as apples, oranges, bananas, frozen and canned fruit, fresh, frozen, and canned vegetables, canned tuna in water, low sugar/high fiber cereals, 100% whole wheat bread, plain hot cereal, pretzels, baked chips, reduced-fat chips, dried fruit, nuts or seeds, cooking spray, grilled chicken, grilled seafood, fruit and vegetable as side dishes, deli sandwich, tacos, yogurt, and granola. Similar to the caregiver score, we assigned one point to each food/beverage item the child reported purchasing in the past seven days, or 0 if they did not purchase that item. Healthy food purchasing score ranged from 0 to 34, mean 5.6, SD 6.9, Cronbach’s alpha: 0.89. 

An Intervention Exposure Score was developed from the Intervention Exposure Questionnaire (IEQ) collected as part of the post-intervention assessment for children and caregivers in the intervention and comparison groups. Participants were asked whether they had ever seen the materials (BHCK logos, posters, handouts, giveaways, educational displays, store shelf-labels) or had purchased food at BHCK corner stores or carryouts. Respondents who answered positively to >3 (half or more) of the red-herring questions, were excluded from analysis. In total, two children and no adult caregivers were excluded due to red-herring questions. 

We calculated exposure scores for each intervention material or activity part of the BHCK corner store and carryout program. Detailed coding of exposure scores is presented in the Supplementary Table S1. Using methods similar to those previously published [25], overall exposure score was developed by summing the re-scaled exposure scores of the various BHCK intervention materials and activities used in corner stores and carryouts (range 0–5.7 points for caregivers and 0–6.7 for children—denoting that a 1-unit change in exposure represent a substantial difference in exposure to intervention activities). The corner store exposure score only included questions pertinent to corner stores (excluded “seeing menu and purchased promoted food from carryouts”), ranged from 0–4.6 (mean: 0.82, SD: 0.9) for caregiver and ranged 0–5.4 (mean: 0.85, SD 1.0) for children. The carryout exposure score only excluded the question about purchasing in a BHCK corner store, and ranged from 0–5.6 (mean: 0.9, SD 1.0) for caregivers and ranged 0–6.1 (mean: 0.89, SD 1.1) for children. Four children and six caregivers had missing information for at least one exposure variable, and were not included in the analyses. The final analytic sample of caregivers was 387 and of children was 385.

The exposure level was stratified by quartiles (very low, low, medium, and high), where we interpret the increase in each quartile as a higher level of exposure to BHCK activities. Caregiver and child exposure scores are found in Supplementary Tables S2 and S3, respectively.

#### 2.2.4. Data Collection and Management

All interviews were conducted in person at a location that was convenient for the participant such as the recreation center, a community location, the participant’s home, or at the Johns Hopkins Bloomberg School of Public Health. Adult caregivers were interviewed for about 90 min at pre- and post-intervention and received $20 gift card for each completed interview. The child participants were interviewed for about 105 min (1 h 45 min) at pre-intervention and post-intervention and received $30 gift card upon completion of the interview. Data collectors were trained intensively, including in-class didactic lectures and practice, role-playing, observation, and feedback from senior level staff. Following the interviews, data were checked for errors by the interviewer and a second research assistant. The data manager ensured that questionnaires had no missing pages and entered the data into Microsoft Access (Microsoft Corp., Redmond, WA, USA). This study was approved by the Johns Hopkins Bloomberg School of Public Health Institutional Review Board (IRB No. 00004203).

### 2.3. Statistical Analysis

#### 2.3.1. Wholesaler

Sales of promoted foods and beverages are presented in the form of a chart, depicting unit sales over time. We also explored the associations between change in promoted food purchasing between wave 1 (July 2014–March 2015) and wave 2 (October 2016–September 2017) of the BHCK intervention. Time ranges for wave 1 and wave 2 were based on the cutoffs for the quarterly data points. Products (such as low-fat mayonnaise and whole wheat pasta) were excluded from the analysis because they were not stocked throughout the entire intervention period or did not have complete data points. 

#### 2.3.2. Corner Stores and Carryouts

All analyses for the corner store and carryout component were conducted using STATA 14.1 (Stata Corp., College Station, TX, USA). We used a two-tailed *t*-test to observe if there was a statistically significant difference in the average HFAI score change of the intervention and comparison corner stores from baseline to post intervention. This was the same method used for carryouts. Because the intervention was implemented in two waves, with the second wave taking into account lessons-learned from the first wave, we also used two-sided *t*-tests to compare the average HFAI scores for comparison and intervention store in each wave. Because of the small sample size of carryouts (*n* = 30) we did not look at the differences by wave, just overall. A two-tailed *t*-test was also used to observe if there was a statistically significant difference in the average weekly change in sales of promoted foods from baseline to post-intervention in the intervention and comparison corner stores.

#### 2.3.3. Child-Caregiver Dyads

We explored associations between change in healthy food purchasing score from baseline to post-intervention and levels of exposure to the BHCK trial among children and caregivers. Assumptions of normality were investigated for each bivariate model using exposure score to BHCK and change in healthy food purchasing score as continuous variables. Based on the Shapiro-Wilk test and on the standardized normal probability plot and quintile-normal plot, the distribution of the residuals was deemed non-normal mainly due to the over dispersion of the outcome and its positively skewed shape (see Supplementary Tables S4 and S5 for sensitivity analyses using multiple linear regression). We used multiple Poisson regression models with robust error variances to estimate the cumulative incidence risk ratio (IRR) for improving healthy food purchasing, given the high prevalence of individuals positively improving healthy food purchasing behavior (>20%). We conducted separate models exploring the change in healthy food purchasing in the overall sample and by stratifying the analyses by intervention and comparison groups. We regressed the outcome on the combined exposure score (corner store & carryout), on the corner store only exposure score, and on the carryout only exposure score. For the caregiver models, we controlled all analyses for caregiver’s age, sex, and education level, household size, and Supplemental Assistance Nutrition Program (SNAP) recipient in the past year. For each child model, we controlled the analysis for child’s age and sex, caregiver’s age, sex, and education level, household size, and Supplemental Assistance Nutrition Program (SNAP) recipient in the past year. To avoid multiple comparison issues, we performed bootstrapping with 2000 iterations, and examined biases adjusted confidence intervals in all regressions to control the proportion of Type I errors. All confidence intervals and standard error remained significant. For all analyses, statistical significance was defined by a *p*-value of <0.05. Future analyses will examine impact of the program on child diet and body mass index (BMI).

## 3. Results

### 3.1. Impact on Sales of Promoted Foods at the Wholesaler Level

We observed overall increases in the sales of promoted beverages and snacks over the course of the two wave BHCK intervention trial (Figure 1). There is an observed overall increase in the number of promoted healthier drinks sold throughout wave 1, and similarly in wave 2. The movement for the promoted snacks was almost stable, with a small increase. By the end of wave 2, there were about 100 more snack units that were sold as compared to baseline. There was an increase in healthier snacks sold from January–March 2016, which was around the time of the snack phase in wave 2. No changes in sales of low-fat cooking ingredients was observed at the wholesaler level.

### 3.2. Impact on Stocking and Sales of Promoted Foods in Corner Stores and Carryouts

The BHCK intervention was associated with a significant increase in stocking of promoted foods and beverages (*p* < 0.01) (Table 3). When broken down by intervention phase, significant changes in the HFAI score were limited to the beverage and snack phases, with a trend observed for low-fat cooking ingredients. Most of the significant changes appeared to take place in wave 2 stores. 

We found an overall average increase in the HFAI for both intervention and comparison carryouts—although intervention carryouts showed a non-significant increase over time (*p* = 0.24). No impact was observed on reported weekly sales of promoted items in corner stores. There was a decrease in the total weekly sales of promoted items for both intervention and comparison stores, although a smaller decrease for intervention stores. This change was not significant (*p* = 0.45). The average change in weekly sales for each sub-total were also non-significant (health beverages *p* = 0.29, healthy snacks *p* = 0.38, and cooking *p* = 0.65). 

### 3.3. Association between Exposure to BHCK Corner Store and Carryout Intervention and Change in Purchasing of Promoted Foods and Beverages among Adult Caregivers

We did not find any statistically significant correlation between positive change in healthy food purchasing behavior and levels of exposure to the BHCK trial among adult caregivers (Table 4). A similar lack of impact was found in our sensitivity analyses (Supplementary Table S4). 

### 3.4. Association between Exposure to BHCK Corner Store and Carryout Intervention and Change in Purchasing Promoted Foods and Beverages among Children

We observed a positive association between healthy food purchasing and exposure levels among children when controlling for child’s age and sex, caregiver’s age, sex, and education level, household size, and Supplemental Assistance Nutrition Program (SNAP) recipient in the multiple Poisson Robust model (Table 5). Children who were highly exposed to the BHCK corner store and carryout intervention had a 24% increased change in healthy food purchasing score, when compared to those in the very low exposure score category (IRR 1.24; 95% CI: 1.02; 1.52). Similar relationships were found when looking at the corner store exposure score only and the carryout exposure score only (IRR 1.22; 95% CI 1.01; 1.48 and IRR 1.21; 95% CI: 1.00; 1.47). Furthermore, we found a positive dose-response relationship when exploring the association between change in healthy food purchasing by exposure levels among intervention group only; children had an increased cumulative incidence risk ratio for healthy food purchasing score by exposure levels (combined, corner store only, and carryout only). 

## 4. Discussion

This is the first study to track the impact of a multilevel food system intervention (i.e., wholesaler, small food stores, consumers) on multiple levels of outcomes (i.e., stocking, sales, and purchasing). We observed increases in wholesaler sales of promoted items, significantly increased stocking of most categories of promoted foods and beverages in corner stores, and significantly increased child purchasing of these foods associated with increased exposure to intervention components. Some of these results have been duplicated in previous studies, including improvements in stocking of healthier food in small stores [26], and improvements in food purchasing [22,27]. However, to our knowledge, no previous trial has shown effects at multiple levels of the community food environment. The fact that we observed increase in sales and stocking of the same types of food items over time at the wholesaler and corner store levels suggest that BHCK was successful in modifying the community food environment when employing a multilevel intervention strategy. 

The BHCK trial was not entirely successful, however. We did not find any significant association between exposure to the intervention and adult caregiver healthy food purchasing. The BHCK intervention primarily targeted children through its youth leader, recreation center, corner store, and carryout components. Due to the design of the intervention, adult caregivers had less direct contact with the program. Caregivers were potentially exposed to the intervention through text messaging and social media, and if they frequented BHCK intervention corner stores and carryouts where they may have interacted with program staff or point of purchase promotion materials. Future multilevel trials targeting both children and their parents should consider enhancing opportunities for direct interaction with adults. An example of a childhood obesity prevention trial that successfully involved caregivers was the Switch™ program [28,29]. Although this was a school-based intervention, Switch™ involved adult caregivers through another two different levels: community (involved city stakeholders and social media for a public education intervention to prevent childhood obesity) and family (mailed information, activities, recipes, meal plan, and tips to achieve nutrition and physical activity goals) [28]. 

We did not observe impact on stocking nor sales at the carryout level. However, our previous trial, Baltimore Healthy Carryouts (BHC), found a positive effect on sales of healthier foods and on total revenues in intervention carryouts when compared to control over time [30,31]. Lack of significant impact on sales observed may be related to the self-reported evaluation methods used to document sales. For BHC, we directly measured sales by asking carryout owners to keep every handwritten order ticket, and we tabulated those throughout the intervention. Therefore, we suspect that our simpler pre-post recall assessment, although less burdensome to storeowners, may not have been precise enough to detect changes in sales. 

Although we found that intervention corner stores stocked healthier food items than comparison ones, we did not detect a significant effect on total sales. In our previous Baltimore Healthy Stores trial conducted with 21 small corner stores to improve healthy food availability in low-income areas of Baltimore, we observed a significant impact on sales of promoted foods in intervention stores than comparison stores [22]. However, this positive result may have been due to the repeated measurements when collecting sales data (every two months (five times total)), which may have attenuated error variability, unlike the current BHCK study where sales reports were conducted at just two time points—pre and post only. A common concern stated by storeowners is the potential for a decrease in total sales [32], though the results from other corner store intervention have found that sales of healthier products can increase [27,33], or remain the same during the intervention duration. Future interventions should evaluate the feasibility, acceptability and efficacy of electronic record keeping in small corner stores and carryouts.

Other limitations to the current work exist. Corner store sales data were based on owner/manager self-report, which may have created a response bias. As in other settings, small corner store owners in Baltimore do not keep electronic or written records of their sales leaving few options for obtaining these data. We used data collectors who were not involved in intervention delivery to ameliorate this concern. Wholesaler sales data analyses were limited by the availability of only quarterly data (four data points per year) and lack of a comparison group. The lack of an additional wholesaler to compare movement and sales limited the opportunity for causal inference to be made for this level of the intervention. Future studies working at the food distribution level should consider the inclusion of a comparison wholesaler, although this may require working in multiple cities. Selection bias in terms of retention of child-caregiver dyads is possible, but is ameliorated by having a comparison group of dyads sampled from similar neighborhoods. We found statistically significant differences in terms of caregiver’s age and sex between those dyad members who were retained versus those lost to follow-up, thus those variables were included as covariates in the caregiver model. It is possible that as our measures focused on a subset of promoted foods and beverages, consumers may have substituted promoted foods for other healthy foods—reducing the possibility of seeing overall dietary analyses. Future planned dietary analyses using a quantitative food frequency questionnaire will examine this possibility. Furthermore, associations between levels of exposure and study outcomes are not causal and should be interpreted with caution. A final limitation of the BHCK trial is one of attribution. We are unable to clearly determine which component or components of this multilevel trial were most effective in leading to study effects. Future analyses are planned to use differential exposure data to tease out these impacts, if possible.

## 5. Conclusions

The B’more Healthy Communities for Kids trial was successful at impacting elements of the food system, including wholesalers, corner stores and child purchasing—one of the first such trials to work at multiple levels of the community food environment. Over a decade of previous work in most of the BHCK intervention venues, including recreation centers [34], corner stores [35], and carryouts [31] served as a prerequisite for this complex trial. Our findings imply that multilevel and multicomponent interventions aiming to change multiple components of the urban food environment, can indeed improve both the choices available to communities (access), as well as consumer behavior (demand). 

## Figures and Tables

**Table 1:** BHCK data collection and intervention implementation timeline.

Wave	Child-Caregiver Dyad Data Collection	Wave 1 Implementation (14 Zones)			Child-Caregiver Dyad Data Collection (Wave 1 Post and Wave 2 Baseline)	Wave 2 Implementation (14 Zones)				Child-Caregiver Dyad Data Collection
Phase		Phase 1 Beverages	Phase 2 Snacks	Phase 3 Cooking		Phase 1 Snacks	Phase 2 Cooking	Phase 3 Beverages	Phase 4 Review	
Promoted Products’ Example		1. Water 2. Sugar free tea mixes 3. Low-sugar fruit drinks	1. Low-fat yogurt 2. Low-fat popcorn 3. Fresh fruits 4. Fresh vegetables	1. Low-sugar cereal 2. 100% whole wheat bread 3. Fresh/frozen vegetables		1. Low-fat yogurt 2. Low-fat popcorn 3. Fresh fruits 4. Fresh vegetables	1. Low-sugar cereal 2. 100% whole wheat bread 3. Fresh/frozen vegetables	1. Water 2. Sugar free tea mixes 3. Low-sugar fruit drinks	1. Low-fat yogurt 2. Fresh/frozen vegetables 3. Small fresh vegetables bags	
Start date	June 2013	July 2014	September 2014	November 2014	April 2015	December 2015	March 2016	May 2016	June 2016	August 2016
End date	June 2014	August 2014	October 2014	February 2015	November 2015	February 2016	April 2016	June 2016	July 2016	January 2017

**Table 2:** Baseline sociodemographic characteristics of BHCK child-caregiver dyads respondents.

Baseline Sociodemographic	Intervention		Comparison	
	*n*	Mean (SD) or %	*n*	Mean (SD) or %
Child’s Characteristics				
Age **^1^**	199	11.7 (1.4)	186	11.9 (1.6)
Sex (female) **^2^**	109	54%	90	60%
Caregiver’s Characteristics				
Age **^1^**	197	39.4 (1.2)	188	40.5 (9.8)
Sex (female) **^2^**	187	94.4%	170	90.4%
Race (African American) **^2^**	185	93.4%	174	93.0%
Household’s Characteristics				
Household Size **^1^**	198	4.5 (1.5)	188	4.5 (1.6)
SNAP **^2^**	133	70.7%	141	71.2%
WIC **^2^**	46	23.2%	44	23.4%

Abbreviations: SD = standard deviation; SNAP = Supplemental Nutrition Assistance Program; WIC = The Special Supplemental Nutrition Program for Women, Infants, and Children. **^1^** Two-tailed *t*-test analysis; **^2^** Chi-square test; Means and proportions were not statically different across the groups.

**Table 3:** Average change in corner store healthy food availability index (HFAI).

Average Change in Corner Store HFAI by Treatment Group			
Change in HFAI	Comparison (*n* = 24)	Intervention (*n* = 26)	*p*-Value ^1^
	Mean (±SD)	Mean (±SD)	
Total score	1.67 (5.35)	5.65 (4.95)	**0.01**
Beverage subscore **^2^**	0.17 (1.09)	0.92 (1.16)	**0.02**
Snack subscore **^3^**	0 (1.10)	1.19 (1.81)	**0.01**
Cooking subscore **^4^**	1.5 (4.35)	3.54 (3.87)	0.08
Average Change in Corner Store HFAI by Wave and Treatment Groups			
Total Score			
Wave 1	5.56 (4.28)	7.86 (4.00)	0.20
Wave 2	−0.67 (4.59)	3.08 (4.83)	**0.05**
Beverage subscore **^2^**			
Wave 1	0.56 (1.13)	0.86 (1.03)	0.52
Wave 2	−0.07 (1.03)	1.0 (1.35)	**0.03**
Snack subscore **^3^**			
Wave 1	0.77 (0.97)	1.64 (1.45)	0.13
Wave 2	−0.47 (0.92)	0.67 (2.10)	0.07
Cooking subscore **^4^**			
Wave 1	4.22 (3.31)	5.36 (3.65)	0.46
Wave 2	−0.13 (4.16)	1.42 (3.03)	0.29

Abbreviations: HFAI = healthy food availability index; SD = standard deviation; **^1^** Two-tailed t-test analysis; **^2^** Beverage subscore included: sugar-free drink mixes, low-sugar fruit drinks, 100% juice, bottled water, flavored water zero calories, low calorie sports drink, lower sugar soda, diet soda; **^3^** Snack subscore included: baked chips, pretzels, low-fat low-sugar granola bars, low-fat microwave popcorn, low-fat bagged popcorn, sunflower seeds, other nuts and seeds, reduced-fat string cheese, low-fat, low-sugar yogurt; **^4^** Cooking subscore included: lean lunch meats, low sodium beans, cooking spray, mustard, low-fat margarine/butter, low-fat mayonnaise, whole wheat bread, whole wheat tortillas, brown rice, whole wheat pasta, low-fat milk, low sugar cereal, canned fruit, fresh fruit, fresh vegetables, low-sodium canned vegetables, fruit cups, frozen vegetables; Bolded numbers represent significance at *p*-value < 0.05.

**Table 4:** Change in healthy food purchasing behavior over time by quartile of exposure level among BHCK caregivers (*n* = 387). **^1^**

Change in Food Purchasing Behavior by Exposure Quartiles	Combined Exposure Score		Corner Store Score		Carryout Score	
	IRR (Robust SE)	95% CI	IRR (Robust SE)	95% CI	IRR (Robust SE)	95% CI
*Healthy Food Purchasing ^**2**^*						
Very LowExposure	Reference		Reference		Reference	
Low Exposure	1.07 (0.21)	0.73; 1.58	0.97 (0.19)	0.65; 1.45	1.07 (0.21)	0.89; 1.34
Medium Exposure	0.71 (0.16)	0.45; 1.10	0.77 (0.17)	0.50; 1.20	0.74 (0.17)	0.95; 1.43
High Exposure	0.92 (0.19)	0.61; 1.37	0.99 (0.20)	0.67; 1.48	0.93 (0.19)	1.00; 1.47
*Healthy Food Purchasing ^**2**^ among Intervention*						
Very Low Exposure	Reference		Reference		Reference	
Low Exposure	0.59 (0.24)	0.25; 1.33	0.54 (0.23)	0.23; 1.24	0.67 (0.27)	0.30; 1.48
Medium Exposure	0.54 (0.18)	0.27; 1.07	0.51 (0.18)	0.24; 1.04	0.56 (0.20)	0.27; 1.12
High Exposure	0.67 (0.22)	0.36; 1.27	0.80 (0.25)	0.43; 1.51	0.70 (0.23)	0.37; 1.33
*Healthy Food Purchasing ^**2**^ among Comparison*						
Very Low Exposure	Reference		Reference		Reference	
Low Exposure	1.33 (0.30)	0.85; 2.07	1.25 (0.29)	0.85; 1.29	1.30 (0.30)	0.82; 2.05
Medium Exposure	0.87 (0.31)	0.43; 1.75	1.18 (0.32)	0.56; 1.11	0.97 (0.31)	052; 1.81
High Exposure	1.17 (0.37)	0.63; 2.17	1.07 (0.36)	0.32; 1.05	1.23 (0.38)	0.66; 2.27

Abbreviation: IRR: cumulative incidence risk ratio; SE: standard error; CI: confidence interval; **^1^** Multiple Poisson Regression on BHCK exposure level (quartiles) among children controlling for caregiver’s age, sex, and education level, household size, and Supplemental Assistance Nutrition Program (SNAP) recipient; **^2^** Healthy Food Purchasing: Healthy food (low fat/low sugar) frequency score by variety of food items purchased in the past month, includes: 1% or skim milk, yogurt, diet soda or diet energy drinks, water, 100% fruit juice, sugar free drinks, unsweetened tea, fresh fruits such as apples, oranges, bananas, frozen fruit, fresh and frozen vegetables, canned tuna in water, dried beans, low sugar, high fiber cereals, 100% whole wheat bread, plain hot cereal, pretzels, baked chips, reduced-fat chips, dried fruit, nuts or seeds, reduced fat butter or margarine, cooking spray, lite mayonnaise. No significance was found at *p*-value < 0.05.

**Table 5:** Change in healthy food purchasing behavior over time by quartile of exposure level among BHCK children (*n* = 385). **^1^**

Change in Food Purchasing Behavior by Exposure Quartiles	Combined Exposure Score		Corner Store Score		Carryout Score	
	IRR (Robust SE)	95% CI	IRR (Robust SE)	95% CI	IRR (Robust SE)	95% CI
*Healthy Food Purchasing ^**2**^*						
Very Low Exposure	Reference		Reference		Reference	
Low Exposure	1.12 (0.12)	0.92; 1.40	1.15 (0.11)	0.94; 1.41	1.10 (0.11)	0.89; 1.34
Medium Exposure	**1.23 (0.12)**	**1.01; 1.50**	1.14 (0.12)	0.94; 1.41	1.17 (0.12)	0.95; 1.43
High Exposure	**1.24 (0.12)**	**1.02; 1.52**	**1.22 (0.12)**	**1.01; 1.48**	**1.21 (0.12)**	**1.00; 1.47**
*Healthy Food Purchasing ^**2**^ among Intervention*						
Very Low Exposure	Reference		Reference		Reference	
Low Exposure	**2.36 (0.74)**	**1.27; 4.38**	**2.15 (0.63)**	**1.21; 3.85**	**1.96 (0.57)**	**1.10; 3.49**
Medium Exposure	**2.72 (0.82)**	**1.51; 4.93**	**2.38 (0.67)**	**1.37; 4.13**	**2.29 (0.64)**	**1.32; 3.95**
High Exposure	**2.81 (0.83)**	**1.56; 5.04**	**2.51 (0.70)**	**1.45; 4.34**	**2.35 (0.64)**	**1.37; 4.03**
*Healthy Food Purchasing ^**2**^ among Comparison*						
Very Low Exposure	Reference		Reference		Reference	
Low Exposure	0.96 (0.11)	0.77; 1.21	1.05 (0.11)	0.85; 1.29	1.01 (0.11)	0.81; 1.25
Medium Exposure	0.92 (0.14)	0.68; 1.23	0.79 (0.14)	0.56; 1.11	0.84 (0.13)	0.62; 1.15
High Exposure	0.52 (0.17)	0.27; 1.01	0.58 (0.17)	0.32; 1.05	0.57 (0.18)	0.30; 1.08

Abbreviation: IRR: cumulative incidence risk ratio; SE: standard error; CI: confidence interval; **^1^** Multiple Poisson Regression on BHCK exposure level (quartiles) among children controlling for child’s age and sex, caregiver’s age, sex, and education level, household size, and Supplemental Assistance Nutrition Program (SNAP) recipient; **^2^** Healthy Food Purchasing: Healthy food (low fat/low sugar) frequency score by variety of food items purchased in the past week, includes: 1% or skim milk, diet soda, water, 100% fruit juice, sugar free drinks, fruit flavored water, unsweetened tea, fresh fruits such as apples, oranges, bananas, frozen and canned fruit, fresh, frozen, and canned vegetables, canned tuna in water, low sugar/high fiber cereals, 100% whole wheat bread, hot cereal, pretzels, baked chips, reduced-fat chips, dried fruit, nuts or seeds, cooking spray, grilled chicken, grilled seafood, fruit and vegetable as side dishes, deli sandwich, tacos, yogurt, granola. Bolded numbers represent significance at *p*-value < 0.05.

## Supplementary Materials

The following are available online at www.mdpi.com/1660-4601/14/11/1371/s1, Table S1: Exposure score development by BHCK intervention materials and activities conducted in corner stores and carryouts, Table S2: Caregiver exposure to the B’more healthy communities for kids intervention materials and activities in corner stores and carryouts by intervention groups (*n* = 387), Table S3: Youth exposure to the B’more healthy communities for kids intervention materials and activities in corner stores and carryouts by intervention groups (*n* = 385), Table S4: Sensitivity analysis of the change in healthy food purchasing behavior over time by quartiles of exposure level among BHCK caregiver (*n* = 387), Table S5: Sensitivity analysis of the change in healthy food purchasing behavior over time by quartiles of exposure level among BHCK youth (*n* = 385).
