# Supplementary material for: The Impact of a Multi-Level Multi-Component Childhood Obesity Prevention Intervention on Healthy Food Availability, Sales, and Purchasing in a Low-Income Urban Area
Source: Int J Environ Res Public Health. 2017 Nov 10;14(11):1371. doi: 10.3390/ijerph14111371 (PMC5708010; doi:10.3390/ijerph14111371)
Supplement: Supplementary file 1 [file ijerph-14-01371-s001.zip › ijerph-219186-supplementary-proof.pdf]

Supplementary Materials: The Impact of a Multi-Level Multi-Component Childhood Obesity Prevention Intervention on Healthy Food Availability, Sales, and Purchasing in a Low-Income Urban Area

Joel Gittelsohn [1,*], Angela Trude [1], Lisa Poirier [1], Alexandra Ross [1], Cara Ruggiero [2], Teresa Schwendler [1] and Betsy Anderson Steeves [3]

Table S1. Exposure score development by BHCK intervention materials and activities conducted in corner stores and carryouts.

Intervention Component	Intervention Material or Activity	Coding of Exposure Score
Corner stores and Carryouts	**Seeing BHCK Logo in different places** (stores, recreation centers, carryouts, social media)	None = 0<br>1–2 places = 1.5<br>3–5 places = 4<br>6 or more = 6
Corner stores and Carryouts	**Seeing shelf-label** in different stores (BHCK corner stores and carryouts)	None = 0<br>1–2 places = 1.5<br>3–5 places = 4<br>6 or more = 6
Corner stores and Carryouts	**Taste tests** (10 questions) (and 4 cooking demos at recreation center—applied to child only)	For each taste test:<br>Yes = 1<br>Maybe = 0.5<br>No = 0
Corner stores and Carryouts	**Posters** (10 questions)	For each poster:<br>Yes = 1<br>Maybe = 0.5<br>No = 0
Corner stores and Carryouts	**Handouts** (9 questions)	For each handout:<br>Yes = 1<br>Maybe = 0.5<br>No = 0
Corner stores and Carryouts	**Giveaways** (17 questions)	For each giveaway:<br>Yes = 1<br>Maybe = 0.5<br>No = 0
Corner stores and Carryouts	**Educational Display** (5 questions)	For each display:<br>Yes = 1<br>Maybe = 0.5<br>No = 0
Carryouts only	**Seeing redesigned menu** (8 questions)	For each menu:<br>Yes = 1<br>Maybe = 0.5<br>No = 0
Carryouts only	**Purchased promotional items** in a BHCK carryout in the past 7 days	Continuous variable: total frequency of purchase summed for i) side dish, ii) menu item, and iii) healthy combo meals.
Corner stores only	**Purchased in a BHCK corner store** in the past 7 days	Continuous variable: total frequency of purchase summed for all stores (*n* = 21)
**Overall Exposure Score to BHCK Corner Store & Carryout**		1. Added points within each intervention material/activity according to number of questions<br>2. Re-scaled exposure to material/activity to 0–1 range<br>3. Summed all re-scaled exposure scores

Table S2. Caregiver exposure to the B'more healthy communities for kids intervention materials and activities in corner stores and carryouts by intervention groups (n = 387).

Caregiver Exposure to BHCK Materials and Activities	Range	Intervention Mean ± SD	Comparison Mean ± SD	*p*-Value
Seeing BHCK Logo in different places	0–1	0.31 ± 0.25	0.13 ± 0.20	<0.001
Seeing Shelf-Label in different stores	0–1	0.07 ± 0.20	0.06 ± 0.21	0.7
Posters	0–1	0.13 ± 0.20	0.07 ± 0.14	0.001
Handouts	0–1	0.20 ± 0.27	0.05 ± 0.13	<0.001
Giveaways	0–1	0.22 ± 0.22	0.03 ± 0.10	<0.001
Educational Displays	0–1	0.09 ± 0.18	0.07 ± 0.17	0.3
Seen Redesigned Menus	0–1	0.15 ± 0.16	0.04 ± 0.12	<0.001
Purchased Promoted Foods in BHCK Carryouts	0–1	0.03 ± 0.14	0.01 ± 0.09	0.15
Taste Tests	0–1	0.04 ± 0.12	0.05 ± 0.16	0.6
Purchased in different BHCK corner stores	0–1	0.07 ± 0.18	0.01 ± 0.08	<0.001
BHCK Corner Store & Carryout Exposure Level	0–10	1.34 ± 1.01	0.54 ± 0.87	<0.001
BHCK Corner Store Only Exposure Level	0–8	1.5 ± 0.92	0.48 ± 0.74	<0.001
BHCK Carryout Only Exposure Level	0–9	1.26 ± 1.01	0.53 ± 0.85	<0.001

Abbreviation: SD (Standard Deviation); Statistically significant improvement in mean score from wave 1 to wave 2 (p-value < 0.05).

Table S3. Youth exposure to the B'more healthy communities for kids intervention materials and activities in corner stores and carryouts by intervention groups (n = 385).

Youth Exposure to BHCK Materials and Activities	Range	Intervention Mean ± SD	Comparison Mean ± SD	*p*-Value
Seeing BHCK Logo in different places	0–1	0.24 ± 0.3	0.13 ± 0.20	<0.001
Seeing Shelf-Label in different stores	0–1	0.07 ± 0.20	0.03 ± 0.10	0.005
Posters	0–1	0.15 ± 0.20	0.05 ± 0.10	<0.001
Handouts	0–1	0.16 ± 0.23	0.04 ± 0.12	<0.001
Giveaways	0–1	0.23 ± 0.24	0.06 ± 0.14	<0.001
Educational Displays	0–1	0.17 ± 0.27	0.06 ± 0.19	<0.001
Seen Redesigned Menus	0–1	0.08 ± 0.20	0.04 ± 0.13	0.02
Purchased Promoted Foods in BHCK Carryouts	0–1	0.04 ± 0.16	0.01 ± 0.09	0.07
Taste Tests	0–1	0.12 ± 0.20	0.03 ± 0.02	<0.001
Purchased in different BHCK corner stores	0–1	0.08 ± 0.20	0.02 ± 0.09	0.001
BHCK Corner Store & Carryout Exposure Level	0–10	1.35 ± 1.29	0.51 ± 0.72	<0.001
BHCK Corner Store Only Exposure level	0–8	1.22 ± 1.13	0.44 ± 0.63	<0.001
BHCK Carryout Only Exposure Level	0–9	1.26 ± 1.22	0.48 ± 0.71	<0.001

Abbreviation: SD (Standard Deviation); Statistically significant improvement in mean score from wave 1 to wave 2 (p-value < 0.05).

Table S4. Sensitivity analysis of the change in healthy food purchasing behavior over time by quartiles of exposure level among BHCK caregiver (*n* = 387) [1].

Change in Food Purchasing Behavior by Exposure Quartiles	Combined Exposure Score		Corner Store Score		Carryout Score	
	Beta (Robust SE)	95% CI	Beta (Robust SE)	95% CI	Beta (Robust SE)	95% CI
Frequency of Healthy Food Purchasing [2]						
Very Low Exposure	Reference		Reference		Reference	
Low Exposure	−0.07 (0.59)	−1.23; 1.09	−0.18 (0.58)	−1.34; 0.97	−0.1 (0.59)	−1.28; 1.04
Medium Exposure	−0.59 (0.59)	−1.74; 0.57	−0.57 (0.58)	−1.73; 0.57	−0.61 (0.58)	−1.75; 0.54
High Exposure	−0.47 (0.59)	−1.63; 0.68	−0.15 (0.58)	−1.31; 1.01	−0.44 (0.58)	−1.59; 0.71
Frequency of Healthy Food Purchasing [2] *among Intervention*						
Very Low Exposure	Reference		Reference		Reference	
Low Exposure	−0.78 (1.19)	−3.14; 1.57	−0.88 (1.14)	−3.14; 1.38	−0.92 (1.16)	−3.22; 1.38
Medium Exposure	−0.16 (1.06)	−2.27; 1.94	−0.30 (1.05)	−2.38; 1.78	−0.26 (1.05)	−2.34; 1.81
High Exposure	−0.65 (1.06)	−2.75; 1.45	−0.11 (1.04)	−2.17; 1.94	−0.68 (1.04)	−2.74; 1.36
Frequency of Healthy Food Purchasing [2] *among Comparison*						
Very Low Exposure	Reference		Reference		Reference	
Low Exposure	0.46 (0.69)	−0.89; 1.82	0.51 (0.70)	−0.87; 1.90	0.51 (0.69)	−0.87; 1.88
Medium Exposure	−0.73 (0.91)	−2.52; 1.05	−0.33 (0.84)	−1.98; 1.33	−0.79 (0.85)	−2.47; 0.89
High Exposure	1.30 (0.97)	−0.62; 3.22	1.25 (0.99)	−0.71; 3.22	1.56 (0.99)	−0.39; 3.52

Abbreviations: SE: robust standard error; CI: confidence interval; [1] Multiple Linear Regression on BHCK exposure level (quartiles) among youth controlling for youth's age and sex, caregiver's age, sex, and education level, household size, and Supplemental Assistance Nutrition Program (SNAP) recipient; [2] Healthy Food Purchasing: Healthy food (low fat/low sugar) frequency score by variety of food items purchased in the past month, includes: 1% or skim milk, yogurt, diet soda or diet energy drinks, water, 100% fruit juice, sugar free drinks, unsweetened tea, fresh fruits such as apples, oranges, bananas, frozen fruit, fresh and frozen vegetables, canned tuna in water, dried beans, low sugar, high fiber cereals, 100% whole wheat bread, plain hot cereal, pretzels, baked chips, reduced-fat chips, dried fruit, nuts or seeds, reduced fat butter or margarine, cooking spray, lite mayonnaise. Bolded numbers represent significance at *p*-value < 0.05.

Commented [m1]: Please bolded the numbers.

Table S5. Sensitivity analysis of the change in healthy food purchasing behavior over time by quartiles of exposure level among BHCK youth (*n* = 385) [1].

Change in Food Purchasing Behavior by Exposure Quartiles	Combined Exposure Score		Corner Store Score		Carryout Score	
	Beta (Robust SE)	95% CI	Beta (Robust SE)	95% CI	Beta (Robust SE)	95% CI
Frequency of Healthy Food Purchasing [2]						
Very Low Exposure	Reference		Reference		Reference	
Low Exposure	0.85 (1.2)	−1.52; 3.25	1.31 (1.22)	−1.08; 3.71	0.92 (1.21)	−1.46; 3.31
Medium Exposure	2.16 (1.22)	−0.23; 4.56	1.61 (1.21)	−0.78; 3.98	2.14 (1.23)	−0.28; 4.56
High Exposure	2.07 (1.23)	−0.35; 4.50	**2.65 (1.22)**	**0.24; 5.06**	2.13 (1.23)	−0.28; 4.55
Frequency of Healthy Food Purchasing [2] *among Intervention*						
Very Low Exposure	Reference		Reference		Reference	
Low Exposure	**4.74 (2.02)**	**0.75; 8.73**	**5.30 (2.1)**	**1.21; 9.39**	**4.59 (1.04)**	**0.55; 8.63**
Medium Exposure	**8.34 (1.90)**	**4.58; 12.09**	**8.59 (1.88)**	**4.87; 12.29**	**8.17 (1.94)**	**4.34; 12.01**
High Exposure	**8.01 (1.84)**	**4.36; 11.65**	**9.14 (1.84)**	**5.50; 12.77**	**7.91 (1.85)**	**4.24; 11.56**
Frequency of Healthy Food Purchasing [2] *among Comparison*						
Very Low Exposure	Reference		Reference		Reference	
Low Exposure	0.62 (1.45)	−2.25; 3.49	1.51 (1.44)	−1.33; 4.36	1.08 (1.45)	−1.79; 3.96
Medium Exposure	−1.03 (1.67)	−4.33; 2.27	−2.21 (1.69)	−5.56; 1.13	−1.24 (1.64)	−4.49; 2.01
High Exposure	−4.16 (1.27)	−8.64; 0.33	−3.31 (2.16)	−7.58; 0.95	−3.67 (2.33)	−8.28; 0.93

Abbreviations: SE: robust standard error; CI: confidence interval; [1] Multiple Linear Regression on BHCK exposure level (quartiles) among youth controlling for youth's age and sex, caregiver's age, sex, and education level, household size, and Supplemental Assistance Nutrition Program (SNAP) recipient; [2] Healthy Food Purchasing: Healthy food (low fat/low sugar) frequency score by variety of food items purchased in the past week, includes: 1% or skim milk, diet soda, water, 100% fruit juice, sugar free drinks, fruit flavored water, unsweetened tea, fresh fruits such as apples, oranges, bananas, frozen and canned fruit, fresh, frozen, and canned vegetables, canned tuna in water, low sugar/high fiber cereals, 100% whole wheat bread, hot cereal, pretzels, baked chips, reduced-fat chips, dried fruit, nuts or seeds, cooking spray, grilled chicken, grilled seafood, fruit and vegetable as side dishes, deli sandwich, tacos, yogurt, granola. Bolded numbers represent significance at p-value < 0.05.

© 2017 by the authors; licensee MDPI, Basel, Switzerland. This article is an open access article distributed under the terms and conditions of the Creative Commons by Attribution (CC-BY) license (http://creativecommons.org/licenses/by/4.0/).
